# Effects of various heavy metal nanoparticles on *Enterococcus hirae* and *Escherichia coli* growth and proton-coupled membrane transport

**DOI:** 10.1186/s12951-015-0131-3

**Published:** 2015-10-16

**Authors:** Zaruhi Vardanyan, Vladimir Gevorkyan, Michail Ananyan, Hrachik Vardapetyan, Armen Trchounian

**Affiliations:** Research Institute of Biology, Faculty of Biology, Yerevan State University, 1 A. Manoukian Str., 0025 Yerevan, Armenia; Department of Materials Technology and Structures of Electronic Technique, Institute of Mathematics and High Technologies, Russian-Armenian (Slavonic) University, 123 H. Emin Str., 0051 Yerevan, Armenia; “Nano-industry” Concern, 4 Bardin Str., 1 bulk, 119334 Moscow, Russia; Department of Medicinal Biochemistry and Biotechnology, Institute of Mathematics and High Technologies, Russian-Armenian (Slavonic) University, 123 H. Emin Str., 0051 Yerevan, Armenia; Department of Microbiology, Microbes and Plants Biotechnology, Faculty of Biology, Yerevan State University, 1 A. Manoukian Str., 0025 Yerevan, Armenia

**Keywords:** Nanoparticles, Heavy metals, *Enterococcus hirae*, *Escherichia coli*, Bacterial growth, The F_O_F_1_-ATPase

## Abstract

**Background:**

Due to bacterial resistance to antibiotics there is a need for new antimicrobial agents. In this respect nanoparticles can be used as they have expressed antibacterial activity simultaneously being more reactive compared to their bulk material. The action of zinc (II), titanium (IV), copper (II) and (I) oxides thin films with nanostructured surface and silver nanoscale particles on *Enterococcus hirae* and *Escherichia coli* growth and membrane activity was studied by using microbiological, potentiometric and spectrophotometric methods.

**Results:**

It was revealed that sapphire base plates with deposited ZnO, TiO_2_, CuO and Cu_2_O nanoparticles had no effects neither on *E. hirae* nor *E. coli* growth both on agar plates and in liquid medium. Concentrated Ag nanoparticles colloid solution markedly affected bacterial growth which was expressed by changing growth properties. *E. hirae* was able to grow only at <1:200 dilutions of Ag nanoparticles while *E. coli* grew even at 1:10 dilution. At the same time Ag nanoparticles directly affected membranes, as the F_O_F_1_-ATPase activity and H^+^-coupled transport was changed either (*E. coli* were less susceptible to nanoparticles compared to *E. hirae*). Ag nanoparticles increased H^+^ and K^+^ transport even in the presence of *N,N′*-dicyclohexylcarbodiimide (DCCD), inhibitor of F_O_F_1_. The stoichiometry of DCCD-inhibited ion fluxes was disturbed.

**Conclusions:**

These results point out to distinguishing antibacterial effects of Ag nanoparticles on different bacteria; the difference between effects can be explained by peculiarities in bacterial membrane structure and properties. H^+^-K^+^-exchange disturbance by Ag nanoparticles might be involved in antibacterial effects on *E. hirae*. The role of F_O_F_1_ in antibacterial action of Ag nanoparticles was shown using *atpD* mutant lacked β subunit in F_1_.

## Background

In recent years many researchers have tried to find out new antibacterial agents, as many microorganisms have acquired antibiotic resistance [1]. In general, microorganisms acquire resistance to antibiotics during antibacterial therapy and other application fields and this property becomes inheritable. As a result of that process high-doses of antibiotics are used which are very toxic [2]. Nanoparticles can be used as an alternative to antibiotics as there are a number of advantages [3]. For instance, nanoparticles of metals with the size of 10 nm and less have high reactivity and can react with other substances practically without complementary energy. A share of surface atoms in nanoparticles is considerably greater than in bulk material and increases with reduction of particle size. Chemical bonds of nanoparticles surface atoms are not compensated and it results in appearance of new electrical, chemical, mechanical, toxic and other properties. At the same time the advantages with nanoparticles are their safety and biocompatibility [4].

It is well known that heavy metal ions affect bacterial cells and different mechanisms are proposed for the explanation of such effects. The results obtained in our laboratory suggested that the target for heavy metal ions might be the F_O_F_1_-ATPase in bacterial cells which in turn regulated the growth of bacteria [5, 6]. Moreover the action of metal ions can be direct or can be mediated by redox potential [5, 6]. Such effects are known for silver ions too which can be explained by interaction of metal ions with bacterial cell membrane blocking respiration and electron transfer which in turn collapses proton motive force [7]. The antimicrobial activity of Ag nanoparticles can occur as a result of nanoparticles penetration into the bacteria causing damage of cell membrane [8].The effects of ions and nanoparticles of heavy metals can differ among bacteria and further investigation of the mechanisms of nanoparticles action is needed.

Nowadays nanoparticles research is of great interest because they can be used in various fields as medicine, veterinary, food industry, manufacturing and etc. These materials can be used for beneficial purposes while maintaining initial properties and functions [4].

Susceptibility of different microorganisms to nanoparticles depends on various factors. It is known that the influence of nanoparticles on Gram-positive and Gram–negative bacteria is not the same which can be explained by chemical and structural differences in bacterial cell wall [2]. Another factor can be bacterial growth rate. Slow growing bacteria are less susceptible to nanoparticles as the expression of stress-response genes takes place during the bacterial growth [2, 9]. The exact cellular mechanisms for the effects of nanoparticles are not clear yet but the toxicity of different nanoparticles depends on type of nanoparticles as well as on bacterial strains, concentration of bacteria and nanoparticles, pH, temperature etc. It is suggested that the addition of Ag and CuO nanoparticles to *Bacillus subtilis* growth medium leads to the cell wall damage, disruption of biochemical processes while the nanoparticles of TiO_2_ have no toxicity on this bacterium in dark conditions. In the case of *Pseudomonas aeruginosa* TiO_2_ causes loss of respiratory activity while Ag nanoparticles disturb permeability and cell division [2]. Ag nanoparticles are one of the most promising nanomaterials today as they have high antibacterial activity [10, 11]. The toxicity of Ag depends on size of nanoparticles: small nanoparticles (1–10 nm) are able to pass through bacterial cell wall, while larger nanoparticles not [7, 12, 13]. By attaching to bacterial cell wall Ag nanoparticles change the permeability of membrane and inhibit cell respiration [12–14]. At the same time, these nanoparticles are non-toxic at low concentrations for human cells [15]. There is no clear information about the targets and the mechanisms of the nanostructures and nanoparticles effects on *Enterococcus hirae.* These bacteria have antibacterial activity (against other bacteria) [16], are used in food industry and could be added as bio-preservatives [17, 18]. They are also used in the production of mixed acids, especially lactic acid [19, 20]. At the same time among enterococci there are pathogenic species which can cause endocarditis, infections of urinary tract and central nervous system [17]. In this respect, it is of significance to study metabolism and behavior of enterococci in the presence of different external factors including heavy metal nanoparticles. Moreover, the effects of nanoparticles can be distinguishing for different bacteria and, therefore, these effects should be further studied and appropriate mechanisms should be revealed.

The aim of this work was to study the action of different materials (such as CuO, TiO_2_ and ZnO) with nanostructured surface and Ag nanoparticles on *Enterococcus hirae* and *Escherichia coli* growth, ATPase activity and proton-coupled ions transport through membrane. It has been shown that the effects were different; they depended on bacterial species and the type of nanoparticles. In addition, it has been determined that the concentrated Ag nanoparticles colloid solution was more effective than TiO_2_ and ZnO thin films with nanostructured surface and microporous Cu_2_O tablet with nanoscale roughness of surface. The changes in ion membrane transport and ATPase activity were established.

## Results

### Effects of TiO_2_ and ZnO thin films with nanostructured surface and microporous Cu_2_O tablet with nano-scale roughness of surface on bacteria

The effects of TiO_2_ and ZnO thin films with nanostructured surface and microporous Cu_2_O tablet with nanoscale roughness of surface on bacteria were determined on agar plates and in liquid growth medium. It was established that none of nanoparticles had any influence on bacterial growth on agar plates. In the case of neither *E. hirae* nor *E. coli* no growth inhibition zones were determined in the presence of all types of nanoparticles. The growth of bacteria was the same as in the case of control sample (clean sapphire substrate without any deposited film). These sapphire substrate had no influence on bacterial growth in liquid growth medium either (no statistically reliable differences were observed compared to control sample, p > 0.05). It was determined that lag phase duration and specific growth rate were the same as in the case of control sample (not shown). These effects did not depend on the volume of growth medium (the effects were studied in different volumes: 15, 10, 5 and 3 ml). No effects were observed even in the case of 3 ml.

### Effects of concentrated Ag nanoparticles colloid solution on bacterial growth

**Fig. 1:**
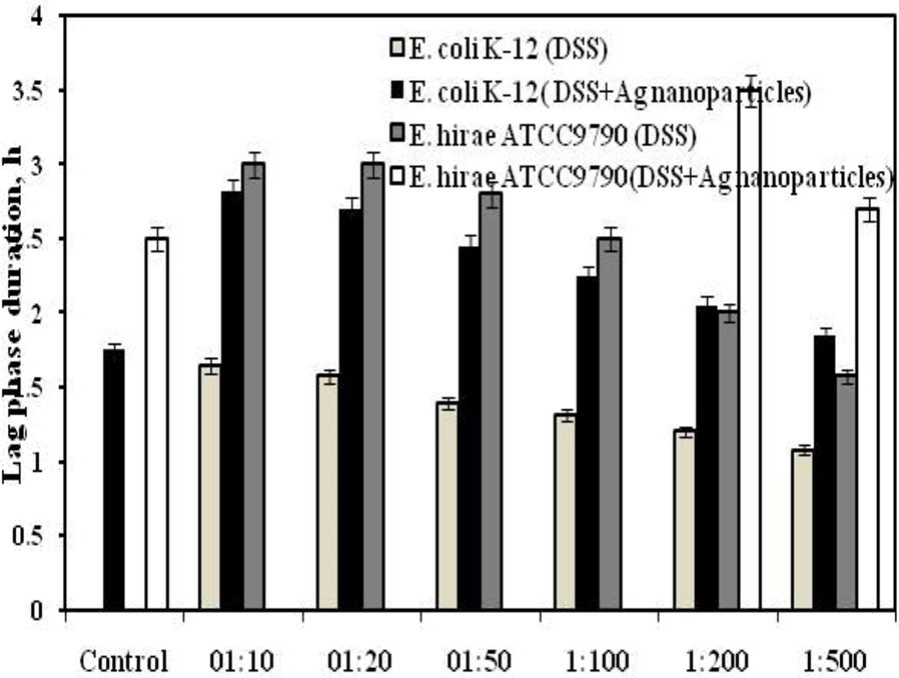
The effect of dioctyl sodium sulfosuccinate (DSS) alone and together with Ag nanoparticles on *E. hirae* ATCC9790 and *E. coli* K-12 lag phase duration. Control was bacterial growth without nanoparticles. Ag nanoparticles and DSS were diluted by 10, 20, 50, 100, 200 and 500 folds. For details see “Methods”

**Fig. 2:**
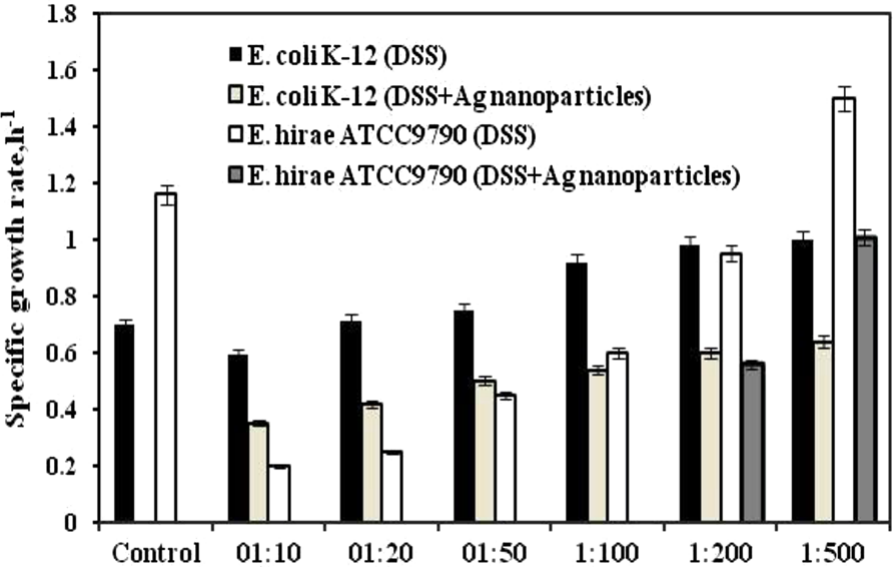
The effect of dioctyl sodium sulfosuccinate (DSS) alone and together with Ag nanoparticles *E. hirae* ATCC9790 and *E. coli* K-12 specific growth rate. For details see “Methods”

The growth of *E. coli* and *E. hirae* was determined in the presence of concentrated Ag nanoparticles diluted by 10; 20; 50; 100; 200 and 500 folds. It was interesting to notice that the effects depended on bacterial species. With *E. hirae* the growth inhibition was stronger as no bacterial growth was detected in the case of 1:10; 1:20; 1:50 and 1:100 dilutions. In the case of 200 and 500 fold dilutions bacteria were able to grow but lag phase duration was prolonged while specific growth rate was decreased (Figs. 1, 2). When concentrated Ag nanoparticles were diluted by 500 fold, the growth was approximately the same as in the control sample (see Figs. 1, 2). The same pattern was observed with the *atp* mutant MS116 either but in the case of the mutant strain the effects were stronger as bacterial growth was detected only with 500 fold dilution of Ag nanoparticles. In contrast to wild type strain, MS116 was not able to grow when nanoparticles were diluted by 200 fold while with 500 fold dilution lag phase duration and specific growth rate was almost the same as in the control sample (not shown).

Interestingly, *E. coli* was able to grow even when concentrated Ag nanoparticles were diluted by tenfold. Lag phase duration was notably prolonged (by ~1.6 fold) while specific growth rate was 2.5- fold lower compared to the control sample (see Figs. 1, 2). These effects had a concentration dependent manner (see Fig. 1). As in the case of *E. hirae* in the presence of Ag nanoparticles diluted by 500 fold the *E. coli* growth was almost the same as in the control sample. It is known that dioctyl sodium sulfosuccinate (DSS) has antibacterial activity especially against Gram-positive bacteria due to its ability to increase the permeability of bacterial cell [21]. To exclude the role of DSS in the inhibition of bacterial growth we have examined the effects of DSS solution on bacterial growth within the same concentration range that was present in concentrated Ag nanoparticles colloid solution (see Materials and methods). As it is shown in Figs. 1 and 2, the solution of DSS had influence both on *E. hirae* and *E. coli* growth but the effects were lower by 1.5–1.7 fold. Moreover it is suggested that in solution, where Ag nanoparticles and DSS are present, synergistic effects can be observed [21].

### Effects of concentrated Ag nanoparticles colloid solution on bacterial proton-coupled membrane transport and ATPase activity

**Fig. 3:**
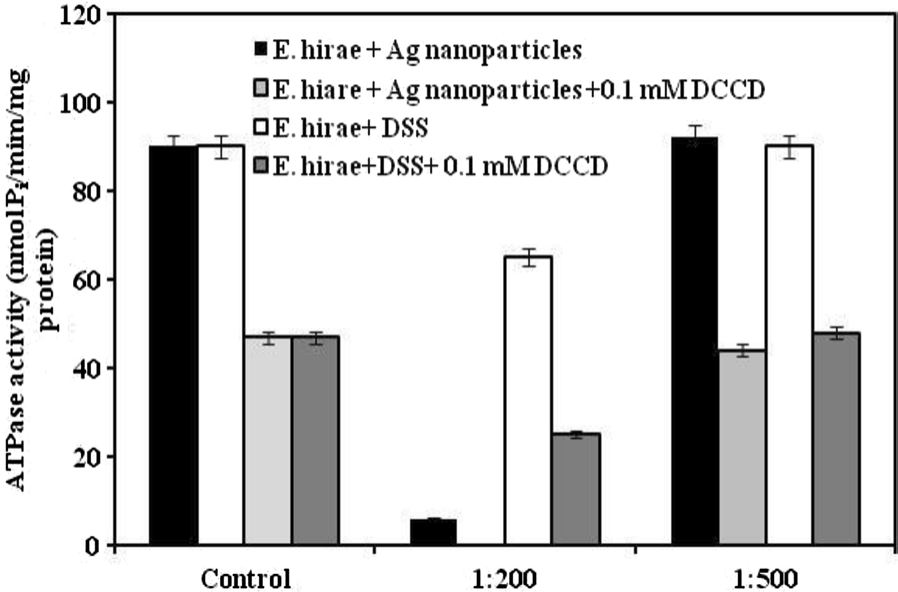
Changes in ATPase activity of membrane vesicles of *E. hirae* in the presence of Ag nanoparticles and DSS in K^+^-containing medium. For details, see “Methods”

As in the case of *E. hirae* bacterial growth was detected only in the case of 1:200 and 1:500 dilutions of Ag nanoparticles, proton-coupled membrane transport and ATPase activity was measured in the presence of these concentrations. It was shown (Fig. 3) that in the case of 1:200 dilution the ATPase activity was lowered by 15 fold compared to the control sample. Moreover when 0.1 mM *N,N′*-dicyclohexylcarbodiimide (DCCD), inhibitor of the F_O_F_1_-ATPase, was added into the assay medium no ATPase activity was detected (see Fig. 3). The results indicate that the F_O_F_1_-ATPase might be a target for Ag nanoparticles in bacterial membrane. As in the case of growth, Ag nanoparticles diluted by 500 fold had no significant effects on ATPase activity as the values were almost the same, as in the control sample (see Fig. 3). For comparison ATPase activity was determined in the presence of DSS either (see Fig. 3). It was shown that in the absence of 0.1 mM DCCD ATPase activity was detected even in the presence of DSS in the case of 100 fold dilution. The values with 200 fold diluted DSS were higher in comparison to the values with Ag nanoparticles in the same concentration (see Fig. 3). The results confirm data observed during bacterial growth, as the effects of Ag nanoparticles were stronger compared to DSS effects. Similar effects were detected with the *atp* mutant MS116 too but the effects were expressed in much less extent (not shown).

The presence of nanoparticles in assay medium led to the increase in H^+^ and K^+^ fluxes (Table 1). More notable effects were with 200 fold dilution when H^+^ and K^+^ fluxes were increased by 2.04 fold and 2.78 fold, respectively (see Table 1). It was revealed that ion fluxes were increased even in the presence of 0.1 mM DCCD, inhibitor of the F_O_F_1_-ATPase [22]. In addition, the ratio (stoichiometry) of DCCD-sensitive H^+^ and K^+^ fluxes was also determined (see Tables 1, 2) which can be indicative for the specific mechanism of H^+^–K^+^ exchange [22, 23]. The ratio was changed depending on concentration of nanoparticles (see Table 1). These results indicate that some disturbance took place after the treatment of bacteria with nanoparticles and the membrane permeability might be changed.

**Table 1:** Proton and potassium ions fluxes across the membrane of *E. hirae* ATCC9790 at the presence of Ag nanoparticles and/or 0.1 mM DCCD

Assay conditions^a^	Ion fluxes (mM/min/10^10^ cells)^b^				
	Total		DCCD-sensitive^c^		
	H^+^	K^+^	H^+^	K^+^	H^+^/K^+^
Control (no additions)	1.32 ± 0.017	0.90 ± 0.0074	0.70 ± 0.019 p^d^ < 0.03	0.31 ± 0.009 p < 0.05	2.2
1:200	2.65 ± 0.007	2.50 ± 0.018	0.85 ± 0.0069	0.55 ± 0.02	0.9
	p < 0.05	p < 0.05	p < 0.02	p < 0.05	
1:500	1.80 ± 0.0085	1.66 ± 0.0081	1.00 ± 0.0075	0.70 ± 0.018	1.3
	p < 0.05	p < 0.05	p < 0.05	p < 0.05	

^a^The bacteria were washed and transferred in Tris–phosphate buffer (pH 8.0) containing 0.4 mM MgSO_4_, 1 mM NaCl, 1 mM KCl; 20 mM glucose was added

^b^Calculated per 10^10^ cells/ml

^c^The difference between fluxes in parallel experiments in the absence and presence of 0.1 mM DCCD

^d^P was calculated for difference between the values of experimental samples and appropriate control

**Table 2:** Proton and potassium ions fluxes across the membrane of *E. coli* K-12 at the presence of Ag nanoparticles and/or 0.1 mM DCCD

Assay conditions^a^	Ion fluxes (mM/min/10^10^ cells)^b^				
	Total		DCCD-sensitive^c^		
	H^+^	K^+^	H^+^	K^+^	H^+^/K^+^
Control (no additions)	2.50 ± 0.016	0.80 ± 0.01	1.10 ± 0.02 p^d^ < 0.05	0.51 ± 0.0084 p < 0.02	2.15
1:10	3.20 ± 0.008	1.35 ± 0.018	1.45 ± 0.01	0.66 ± 0.016	2.2
	p < 0.03	p < 0.05	p < 0.05	p < 0.05	
1:20	3.00 ± 0.01 p < 0.03	1.20 ± 0.016 p < 0.03	1.35 ± 0.0081 p < 0.05	0.61 ± 0.02 p < 0.05	2.2
1:50	2.92 ± 0.02	1.15 ± 0.01	1.30 ± 0.02	0.60 ± 0.01	2.15
	p < 0.04	p < 0.03	p < 0.05	p < 0.05	
1:100	2.80 ± 0.007	1.00 ± 0.02	1.25 ± 0.02	0.57 ± 0.0079	2.2
	p < 0.04	p < 0.04	p < 0.05	p < 0.05	
1:200	2.70 ± 0.02	0.95 ± 0.016	1.25 ± 0.017	0.52 ± 0.015	2.3
	p < 0.05	p < 0.05	p < 0.05	p > 0.05	
1:500	2.55 ± 0.009	0.90 ± 0.0065	1.20 ± 0.02	0.52 ± 0.02	2.3
	p > 0.05	p < 0.05	p > 0.05	p > 0.05	

^a^The bacteria were washed and transferred in Tris–phosphate buffer (pH 8.0) containing 0.4 mM MgSO_4_, 1 mM NaCl, 1 mM KCl; 20 mM glucose was added

^b^Calculated per 10^10^ cells/ml

^c^The difference between fluxes in parallel experiments in the absence and presence of 0.1 mM DCCD

^d^P was calculated for difference between the values of experimental samples and appropriate control

With *E. coli* similar effects were detected but with less extent: H^+^ and K^+^ fluxes were increased too but the effects were weaker. Ions fluxes were detected even in the case of 1:10, 1:50 and 1:100 dilutions of Ag nanoparticles concentration and in the presence of 0.1 mM DCCD (Table 2). The effects had a concentration dependent manner and the ratio of DCCD-inhibited fluxes was fixed (see Table 2).

## Discussion

Nowadays heavy metal nanoparticles are widely used in various fields including medicine and drug production. Nanoparticles are used as drug carriers which allow delivering drugs directly in the therapy of various tumors, such as breast cancer, lung cancer [24]. As nanoparticles have antibacterial activity they are used during wound healing and postoperative recovery. The use of nanoparticles as immunomodulators is also known in the field of clinical medicine [25]. At the same time the exact mechanisms of action and toxicity of nanoparticles are not known yet. In this respect we have tried to determine effects of different nanoparticles on Gram-positive (*E. hirae*) and Gram-negative (*E. coli*) bacterial strains and found out possible targets in bacterial cells.

As it was mentioned above, the effects of nanoparticles depend on bacterial species (the effects were stronger with *E. hirae*) which can be explained by differences in bacterial cell wall structure, composition of membrane and membrane-associated properties [22, 26]. These results are in accordance with data reported recently [2]. It has been shown that Gram-positive strains show higher susceptibility to nanoparticles than Gram-negative strains [27–29]. Yoon and coauthors [27] determined the susceptibility constants and revealed that *B. subtilis* is more sensitive to Ag nanoparticles compared to *E. coli*. Similar effects were observed by Azam and coauthors [29]. It was shown that Gram-negative *E. coli* and *P. aeruginosa* for both Cu_2_O and ZnO films with nanostructured surfaces had lower inhibition-zone sizes than Gram-positive *B. subtilis* and *S. aureus* [29]. It is also established that the effects of nanoparticles depend on their size, stability and concentration in the growth medium [29]. At the same time the effects depend on type of nanoparticles as it was shown that Ag nanoparticles have stronger bactericidal effect against *E. coli* and *S. aureus* compared to Cu nanoparticles [9]. Similar effects have been observed, as Ag nanoparticles had the strongest effects (see Figs. 1, 2). At the same time Ag nanoparticles were in concentrated colloid solution, and the interaction of nanoparticles with bacterial cells was easier compared to ZnO and TiO_2_ thin films and microporous Cu_2_O. Moreover separate addition of DSS which was used as a surfactant and stabilizer in Ag nanoparticles colloid solution caused an inhibition in bacterial growth but the effect was lowered by 1.7 fold compared to colloid solution (see Figs. 1, 2). These results indicate the specific action of Ag nanoparticles. The effects of Ag nanoparticles on *E. hirae* were investigated by Manivasagam et al. [8] using well-diffusion method; the other methods are required. However, differences between the effects on various bacteria should be clarified and mechanisms of action are not known yet.

It is known that Ag has been used in biotechnology and many fields as medicine, veterinary and cosmetics, as a water disinfection agent, in jewelry production as an antimicrobial and antifungal agent [30]. When this metal is prepared as a nanoparticle with sizes of 10–12 nm the antimicrobial effect is better as they have larger specific surface area, as suggested [27]. It is also proposed that Ag nanoparticles are able to interact with bacterial membranes increasing permeability, changing structure of membranes and finally leading to cell death [31]. After penetrating through bacterial membrane Ag nanoparticles are able to damage DNA or inactivate enzymes [32, 33]. As it was determined [1], Ag nanoparticles are more toxic for microorganisms compared to other metals simultaneously they are non-toxic for human cells at low concentrations [34]. We have shown that Ag nanoparticles affect not only the growth of both *E. hirae* and *E. coli* (see Figs. 1, 2) but H^+^ and K^+^ fluxes were changed in the presence of these nanoparticles (see Tables 1, 2).

**Fig. 4:**
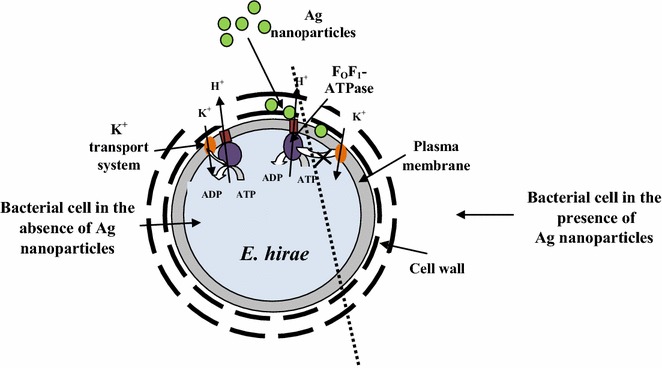
Proposed scheme for Ag nanoparticles effects on bacteria. Ag nanoparticles are suggested to affect H^+^-coupled membrane transport of bacteria. *E. coli* and *E. hirae* possessed H^+^–K^+^ exchange through F_O_F_1_ and K^+^-transport system (*left side*). In the case of *E. hirae* Ag nanoparticles changed the stoichiometry of H^+^–K^+^ exchange through membrane. The effect on F_O_F_1_ and disturbance of the interaction between F_O_F_1_ and K^+^-transport system (*right side*) might be responsible for stronger antibacterial effects with *E. hirae*

It is suggested that membrane-associated ATPase activity of *E. coli* and *E. hirae* and H^+^-coupled K^+^ transport is the result of the F_O_F_1_-ATPase interaction with K^+^ transport system, Trk and KtrI system, respectively [22, 23, 35, 36]. Ion fluxes increased even in the presence of DCCD were indicating that Ag nanoparticles affect bacterial membrane leading to changes in structure and permeability. These effects depend on bacterial species either. In the case of *E. coli* the ratio of DCCD-sensitive H^+^–K^+^ exchange was fixed (see Table 2) while with *E. hirae* the stoichiometry was changed indicating that the interaction between the F_O_F_1_-ATPase and KtrI system was disturbed (Fig. 4). Such effects can explain the stronger effects with *E. hirae* compared to *E. coli*. It was shown that Ag nanoparticles directly affect the F_O_F_1_-ATPase as this ATPase activity was changed even in the absence of DCCD and the effects were stronger with wild-type strain. DSS affected ATPase activity either but the values were higher. Ag nanoparticles might affect the interaction of the F_O_F_1_-ATPase with secondary transport systems or can directly affect ATPase (see Fig. 4). The data with *E. hirae* mutant MS116 confirmed this suggestion, as the results were similar to the effects with wild-type strain but were expressed in much less content. Such differences might be connected with the F_O_F_1_-ATPase which is defective in this strain. There are a lot of studies confirming the fact that ATPase can be a target for several external factors, such as antibiotics and heavy metals [5, 6, 37]. Similar effects were observed by Chichova et al. with mammalian cells either [38], showing that Ag nanoparticles inhibited mitochondrial ATPase activity of rat liver cells. As the F_O_F_1_-ATPase has a crucial role in cell metabolism, such effects can be defining for bacterial growth and survival.

## Conclusions and significance

The results pointing out the role of the F_O_F_1_-ATPase in bacterial response to Ag nanoparticles are absolutely novel and important especially for *E. hirae*. These findings can be decisive in understanding the mechanisms of the effects of metal nanoparticles on different bacteria. This information might be helpful while using nanoparticles as antibacterial agents in biotechnology and other applications.

## Methods

### Bacterial strains and growth, E_h_

This study was performed with *E. hirae* ATCC9790 wild type strain and the *atpD* mutant strain MS116 (lacked β subunit in F_1_) and *E. coli* K-12 wild-type strains. *E. hirae* was supplied by Prof. H. Kobayashi (Graduate School of Pharmaceutical Sciences, Chiba University, Chiba 263, Japan) and Prof. M. Solioz (Department of Clinical Pharmacology, University of Bern, Bern 3010, Switzerland) [22]. *E. coli* was laboratory stock strain.

*E. hirae* was grown under anaerobic conditions 37 °C in the medium that contains 1 % tryptone, 0.5 % yeast extract, 1 % K_2_HPO_4_ with addition of 0.2 % glucose at pH 8.0 [22, 23]. *E. coli* was grown in peptone (2 % peptone, 0.5 % NaCl) medium buffered with 0.1 M K_2_HPO_4_ (pH 7.5), 0.2 % glucose was added [39]. The pH of the medium was measured with pH-selective electrode (HJ1131B, Hanna Instruments, Portugal) and adjusted by 0.1 M NaOH or HCl. The bacterial growth rate was determined by measuring the changes in optical density (OD) of bacterial suspension using a spectrophotometer (Spectro UV–vis Auto, Labomed, USA) at a wave length of 600 nm. Bacterial growth was monitored every hour till 8 h and at 24 h. The bacterial suspension was washed and concentrated by centrifugation at 3600*g* for 15 min and transferred into appropriate medium.

The latent (lag) phase duration was determined as described previously [5]. The specific growth rate was calculated by dividing 0.693 (lg2 = 0.693) by the doubling time of OD in the ranges where the changes in the logarithm of OD depended on time in a linear manner.

### Nanoparticles susceptibility

**Fig. 5:**
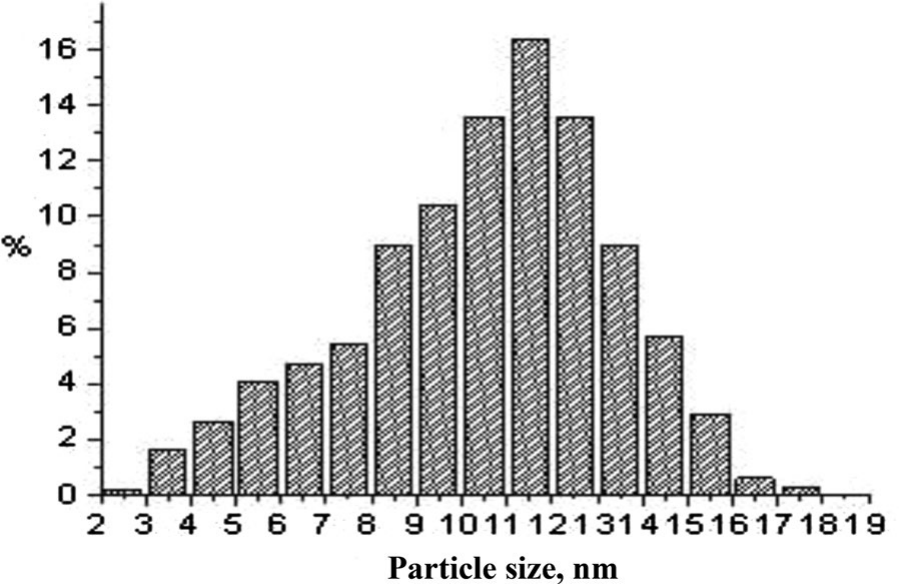
Ag nanoparticles distribution in the colloid solution depending on their size. For source of Ag nanoparticles, see “Methods”

TiO_2_ and ZnO thin films with nanostructured surface deposited on sapphire substrate, microporous Cu_2_O tablet with nano-scale roughness of surface and concentrated Ag nanoparticles colloid solution were used. As a colloid solution of Ag nanoparticles we used “Biocidal Additive” produced by Concern “Nano-industry” (Moscow, Russia). Ag nanoparticles had sizes in the range from 3 to 15 nm; optimal sizes were of 10–12 nm (Fig. 5).

This water solution consists of 0.21 mg/cm^3^ Ag nanoparticles and 18.75 mg/cm^3^ DSS.The latter is an anionic surfactant, a substance that lowers the surface tension of water and it is found that good water solubilizing capacity is depended on its structure [40].

The microporous Cu_2_O tablets were prepared by pressing of CuO micro-scale (1–10 μm) powder and posterior annealing during 3 h at 800 °C in the ambient of oxygen at 1 mbar pressure. In accordance with Cu–O phase diagram [41] at these annealing conditions due to the chemical reaction 4CuO → 2Cu_2_O + O_2_ the molecules of CuO loses oxygen and transforms into Cu_2_O. This phenomenon results in formation of nanoscale roughness on the surface of Cu_2_O film in the range ±500 nm [42]. Susceptibility of microorganisms to nanoparticles was studied by determining bacterial growth in the presence of the nanoparticles. Sapphire base plates with deposited nanoparticles and concentrated Ag nanoparticles colloid solution were added both in bacterial growth medium where lag phase duration and specific growth rate were determined [37] and on agar plates [43]. As in the case of antibiotics, susceptibility of bacteria to nanoparticles was observed by determination of standardized agar diffusion zones (halos) indicating about bacterial growth inhibition. Bacterial suspension of 100 µl was disseminated on plates; disks with deposited nanoparticles were placed on agar and incubated in 37 °C for 24 h. After the incubation agar halos were observed and diameters were measured.

### ATPase assay

ATPase activity of membrane vesicles was measured by amount of liberated inorganic phosphate (P_i_) after adding 5 mM ATP by a spectrophotometric method [44, 45]. The assay mixture was 50 mM Tris–HCl (pH 8.0), containing 0.4 mM MgSO_4_ and 100 mM KCl. When it was necessary, membrane vesicles were pre-incubated with nanoparticles or DCCD for 10 min. The corrections were made for blanks without ATP or membrane vesicles. Relative ATPase activity was expressed in nmol P_i_ per mg protein in 1 min. Membrane vesicles were isolated as described earlier [46] except that the buffers lacked K^+^.

### Proton-potassium exchange assays

Transport of H^+^ and K^+^ through the membrane in the whole cells was assayed by monitoring changes in their activity in the medium using appropriate selective electrodes (HJ1131B, Hanna Instruments, Portugal, and PVC membrane type, Cole Parmer Instruments Co., USA) as described elsewhere [22, 36]. Ions fluxes are expressed as the change in external activity of the ion in mM/min/10^10^ cells in a unit of medium volume (ml). Electrode readings data were outputted automatically by LabView computer program (National Instruments Co., USA). Using this program, electrode readings were calibrated by titration the assay medium (200 mM Tris–phosphate buffer (pH 8.0) containing 0.4 mM MgSO_4_, 1 mM NaCl and 1 mM KCl) with 0.01 N HCl and 0.02 mM KCl. When mentioned, cells were treated with metal nanoparticles and/or DCCD (0.1 mM) for 10 min prior assays. Preparation of whole cells for determination of ion fluxes was described before [22].

### Data processing and reagents

The average data are presented from 3 independent measurements. The standard errors calculated using Microsoft Excel 2013 do not exceed 3 % (if not mentioned). The validity of the differences between the changes obtained and the controls are estimated by Student P value: if there is no other value, then p < 0.01.

Glucose (Borisov Plant of Medicinal Preparations, Belarus), agar, DCCD (“Sigma”, USA), tryptone, yeast extract, Tris (amino-methane) (“Carl Roth GmbH & Co”, Germany) as well as the other reagents of analytical grade were used in the study.

## Abbreviatons

DCCD: *N,N′*-dicyclohexylcarbodiimide
DSS: dioctyl sodium sulfosuccinate
F_O_F_1_-ATPase: proton translocating ATPase
